# Fabrication and Characterization of Calcium-Phosphate Lipid System for Potential Dental Application

**DOI:** 10.3389/fchem.2020.00161

**Published:** 2020-03-25

**Authors:** Ningxin Zhu, Dan Wang, Fei Xie, Man Qin, Zhiqiang Lin, Yuanyuan Wang

**Affiliations:** ^1^Department of Pediatric Dentistry, School and Hospital of Stomatology, Peking University, Beijing, China; ^2^Beijing Key Laboratory of Tumor Systems Biology, School of Basic Medical Sciences, Institute of Systems Biomedicine, Peking University Health Science Center, Beijing, China

**Keywords:** calcium-phosphate lipid, dental pulp stem cells, biocompatibility, LPS, anti-inflammation

## Abstract

Lipid has been widely studied as a vehicle and loading vector, but there have been no reports of any such related application in the dental field. The purpose of this research was to fabricate and characterize a nano-size calcium-phosphate lipid (CL) system as a potential vehicle in dental regeneration study, wherein the biocompatibility with dental pulp stem cells (DPSCs) was evaluated. The effect of CL on DPSCs proliferation was analyzed by a CCK-8 assay, and the anti-inflammatory effect was investigated by quantitative polymerase chain reaction (qPCR). Moreover, the effect of CL on odontogenic differentiation of inflamed DPSCs (iDPSCs) was studied by Alizarin red staining, tissue-non-specific alkaline phosphatase (TNAP) staining, qPCR, and western blot analyses. The results of this study showed that CL did not affect the proliferation of DPSCs, it down-regulated the inflammatory-associated markers (*IL-1*β*, IL-6, TNF-*α*, COX-2*) of DPSCs treated with *Escherichia coli* lipopolysaccharide (LPS), and enhanced the *in-vitro* odontogenic differentiation potential of iDPSCs. This novel biomaterial has a broad application prospect for its bioactivity and flexible physical property, and thus represents a promising pulpal regeneration material.

## Introduction

The calcium-phosphate lipid system has shown success in a wide range of treatment strategies due to its multiple properties, such as an efficient encapsulating ability and its antimicrobial properties (Verderosa et al., 2019), many of which were yet unexplored (Satterlee and Huang, 2016). Since 1970s, an attempt to characterize the calcium-phosphate complex to increase transfection efficiency and allow delivery has been made (Graham and van der Eb, 1973; Maitra, 2005; Sokolova et al., 2006; Xu et al., 2019). Lipids have broad application prospects since they were easily designed, synthesized, and characterized (Zhi et al., 2018; Williams and Grant, 2019). Many factors on the surface of the nanoparticle can influence blood residence time and organ-specific accumulation (Alexis et al., 2008; Nuti et al., 2018; Tyo et al., 2019). The length and type of aliphatic chain determine the phase transition temperature and the fluidity of the bilayer, resulting in the stability, and transfection efficiency of a given lipid (Zhi et al., 2018; Li et al., 2019). In 2010, calcium phosphate nanoparticles were successfully coated with a lipid bilayer (Li et al., 2010), and these so-called Lipid-Calcium-Phosphate (LCP) nanoparticles showed efficient gene silencing ability *in vivo* (Li et al., 2012) as well as versatility in encapsulating various therapeutic compounds, such as small molecule drugs (Zhang et al., 2014), siRNA (Yang et al., 2012; Yao et al., 2013), and peptides (Xu et al., 2013). In consideration of its latent capacity, there is a great application possibility to tissue engineering. The DNA-lipid film was studied as a bone-guiding scaffold in craniofacial tissue (Fukushima et al., 2004), however, there has been little research concerning lipid-based pulp tissue regeneration.

Pulpal vitality of the young immature tooth is essential for root development and reparative dentin formation, which is easily affected, when exposed to external cues. Irreversible pulpitis in an immature permanent tooth will interrupt root development, causing worse function, and shorter survival time of the tooth. Dental pulps with pulpitis suffer higher expressions of pro-inflammatory cytokines (IL-1α, IL-1β, IL-6, and TNF-α) and innate immune response (TLR2, TLR4) than pulps without pulpitis (Zhai et al., 2019). It is a great challenge to find a bioactive and anti-inflammatory material for dental pulp regeneration if failed to preserve vital pulp tissue. The purpose of this novel study is to fabricate the calcium-phosphate lipid (CL) suspension and evaluate its biocompatibility as a composition of dental pulp tissue engineering material.

## Materials and Methods

### Synthesis of Calcium-Phosphate Lipid (CL) and Structural Characterization

**Figure 1:**
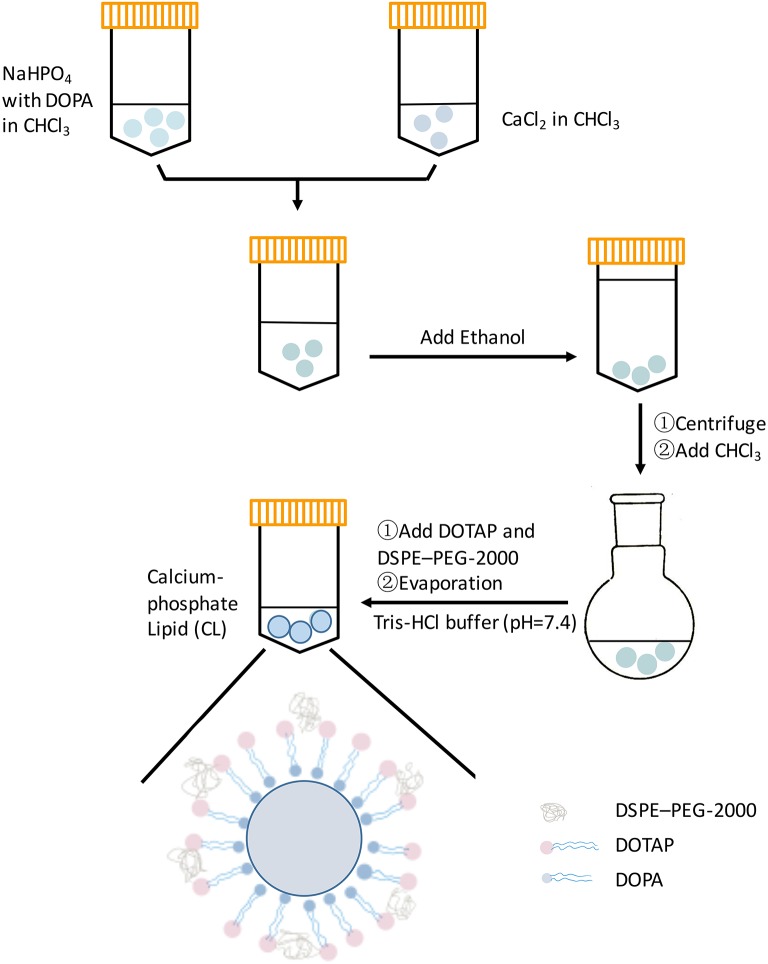
Schematic representation of proposed method to synthesis CL.

The fabrication of CL was performed as previously reported (Li et al., 2012). Briefly, 300 μL of 500 mM CaCl_2_ was dispersed in 15 mL Cyclohexane/Igepal CO-520 (71/29 v/v) solution to form a very well-dispersed water-in-oil reverse micro-emulsion. Three hundred microliter of 25 mM Na_2_HPO_4_ (pH = 9.0) in chloroform was dispersed in the same solution to form the phosphate phase, and 200 μL (20 mg/mL) dioleoylphosphatydicacid (DOPA) in chloroform was added. The amphiphilic DOPA could stay at the interface of micro-emulsions and interact with the precipitated CaP core through binding with the surface Ca^2+^ (Kord Forooshani et al., 2019). The above two solutions were mixed adequately for 20 min, then 30 mL ethanol was used to wash the cyclohexane and surfactant 2–3 times (centrifuging at 12,000 g for 15 min each time). The pellets were dissolved in 1 mL chloroform forming CaP core suspension. Then 500 μL of CaP core was mixed with 50 μL of 10 mM DOTAP/Cholesterol (1:1) and 50 μL of 3 mM DSPE–PEG-2000 to encapsulate the core. After evaporating the chloroform, the residual lipid was dispersed in 400 μL of 5 mM Tris-HCl buffer (pH = 7.4) to form calcium-phosphate lipid (Figure 1). The CL suspension was diluted with α-modified minimum essential medium (α-MEM, GIBCO/BRL, USA) to obtain the gradient solutions (50μg/mL, 5μg/mL, 500ng/mL).

The surface morphology of CL was investigated by scanning electron microscopy (SEM) analysis (JSM 7900F, Japan). The hydrodynamic diameter of CL was determined at room temperature using a dynamic light scattering device (DLS, Zetasizer Nano, Malvern, UK). The calcium content was determined through Inductively Coupled Plasma-Atomic Emission Spectrometry (ICP-AES) and the sample was diluted with distilled de-ionized water (DD water) and phosphate buffer saline (PBS) in 1:1 v%.

### Cell Culture

This research had been reviewed and approved by the Ethics Committee of the Peking University Health Science Center. The human dental pulp stem cells (DPSCs) were collected from 14- to 18-year-old patients who were undergoing dental extraction as part of orthodontic treatment. DPSCs were isolated from the pulp tissue derived from the root canal and then digested in 4 mg/mL dispase (Sigma-Aldrich, St. Louis, MO, USA) and 3 mg/mL type-I collagenase (Sigma-Aldrich, St. Louis, MO, USA) for 1 h at 37°C. Single-cell suspensions were obtained by passing cells through a 70-μm strainer (Falcon; BD Biosciences, San Jose, CA). The cell suspensions (0.5–1.0 × 10^3^/well) were seeded on 12-well plates containing α-MEM supplemented with 10% fetal bovine serum (FBS, GIBCO, USA), 100 U/mL penicillin, and 100 μg/mL streptomycin and then incubated at 37°C in 5% CO_2_. DPSCs were identified by our previously published method (Wang et al., 2013). DPSCs between the fourth and sixth passage were used for this research.

### Quantitative Polymerase Chain Reaction Analysis (qPCR)

The qPCR analysis was taken out by following a previously published method. In brief, *Escherichia coli* lipopolysaccharide (LPS) (Sigma Aldrich, St Louis, MO, USA) powder was dissolved in sterile distilled water to a final concentration of 1 μg/mL. According to the results of our previous work (Zhu et al., 2019), after stimulation of LPS within 6 h, mRNA level of inflammatory cytokines of DPSCs will increase significantly. DPSCs were treated with 1 μg/mL LPS for 1 h to trigger inflammatory reaction, and termed LPS-induced DPSCs (inflamed dental pulp stem cells, iDPSCs). iDPSCs were co-cultured with CL dilutions for 3 h. Untreated DPSCs were used as a control group. iDPSCs group and CL group (iDPSCs + CL dilution) were compared with control group at 3 h. Total RNA were extracted from the treated iDPSCs using TRIzol (Introgen, Carlsbad, CA, USA), then converted to cDNA with Moloney murine leukemia virus reverse transcriptase (M-MLV RTase, Promega, Madison, WI, USA). qPCR analysis was performed on a total volume of 20 μL in SYBR® Green master mix (Rox, Roche Applied Science, IN, USA), with 0.5 μL cDNA and 200 nM of the primers. Specific primers (listed as Table 1) for Glyceraldehyde-3-phosphate dehydrogenase (GAPDH), dentin sialophosphoprotein (DSPP), interleukin-1β (IL-1β), interleukin-6 (IL-6), interleukin-8 (IL-8), tumor necrosis factor-α (TNF-α), cyclooxygenase-2 (COX-2), alkaline phosphatase (ALP), osteocalcin (OCN), runt-related transcription factor 2 (RUNX2), and bone sialoprotein (BSP) were designed by Primer 3 and synthesized (BGI, China). qPCR amplifications were performed as the following thermal cycling conditions: 50°C for 2 min, then 95°C for 10 min, followed by 40 cycles of 94°C for 15 s, and 60°C for 1 min. ABI PRISM 7500 Sequence Detection System (Applied Biosystems, Foster City, CA, USA) was used for the reaction. All data were analyzed by using PRISM6 software (one-way ANOVA and LSD comparison test).

**Table 1:** Primers used for quantitative PCR.

**Target gene**	**Sequence**	**Product size (bp)**
***GAPDH***	Forward: GCAAATTCCATGGCACCGTC	465
	Reverse: GGGGTCATTGATGGCAACAATA	
***DSPP***	Forward: TCCTAGCAAGATCAAATGTGTCAGT	152
	Reverse: CATGCACCAGGACACCACTT	
***BSP***	Forward: ACCCTGCCAAAAGAATGCAG	281
	Reverse: TGCCACTAACATGAGGACGT	
***RUNX2***	Forward: CACTGGCGCTGCAACAAGA	157
	Reverse: CATTCCGGAGCTCAGCAGAATAA	
***IL-1β***	Forward: TGCACGATGCACCTGTACGA	298
	Reverse: AGGCCCAAGGCCACAGGTAT	
***IL-6***	Forward: ACGAACTCCTTCTCCACAAGC	397
	Reverse: CTACATTTGCCGAAGAGCCC	
***COX-2***	Forward: CTGGCGCTCAGCCATACAG	401
	Reverse: ACACTCATACATACACCTCGGT	
***TNF-α***	Forward: CAGAGGGAAGAGTTCCCCAG	285
	Reverse: CCTCAGCTTGAGGGTTTGCTAC	

### Western Blot Analysis

According to the results of qPCR, iDPSCs were treated with CL dilutions for seven days, and cells were lysed in RIPA buffer containing protease and phosphatase inhibitors. Proteins were extracted and then quantified by using the BCA Protein Assay (Pierce, USA). Forty micrograms of proteins derived from each sample were separated on 10% SDS-PAGE gels and transferred to PVDF membranes (Millipore, Bedford, MA, USA) at 100 V for 60 min. The membranes were incubated in blocking buffer (5% non-fat dry milk in Tris-buffered saline containing 0.05% Tween-20, pH 7.4) for 1 h and then incubated with antibodies: DSPP (ab216892, Abcam, China), RUNX2 (D1L7F, Cell Signaling Technology, Danvers, MA, USA), OCN (ab13420, Abcam, China), and β-actin (D6A8, 8457T, Cell Signaling Technology, Danvers, MA, USA) in 1:1000 dilutions at 4°C overnight. The membranes were then incubated with horseradish peroxidase-conjugated secondary antibody (PV9001, PV9002, ZSJQ, China) for 1 h at room temperature. The bands were visualized by using Fusin Fx (Vilber Lourmat, France).

### Statistical Analysis

ImageJ and PRISM6 were used. Statistical analysis was performed using one-way ANOVA and LSD comparison test. The level of statistical significance was *p* < 0.05.

## Results

### The Physical Properties and Biocompatibility of CL

**Figure 2:**
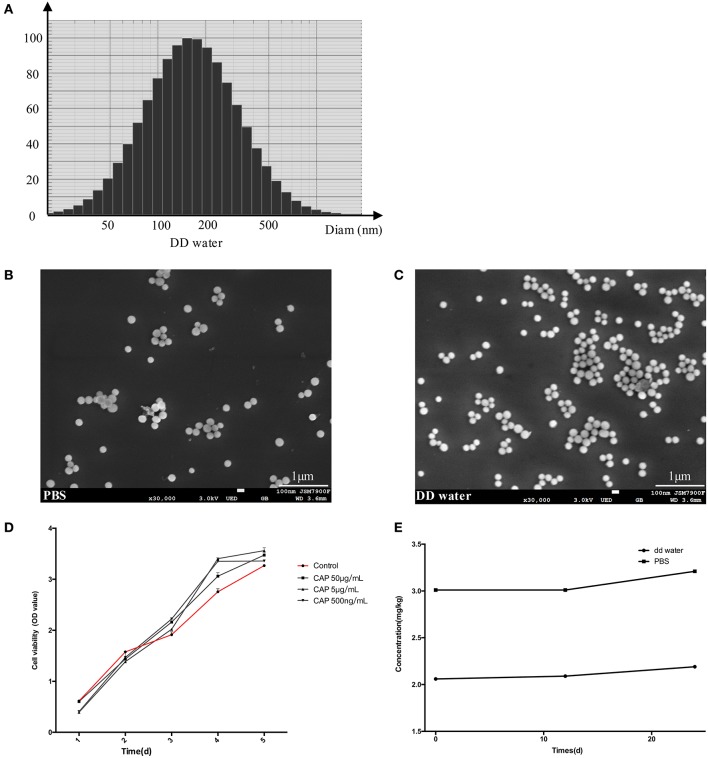
The results of diameter scanning **(A)** and SEM **(B,C)** showed the physical properties of CL, which dispersed better in PBS **(B)** than DD water **(C)**. CCK-8 assay results **(D)** at 1, 3, 5, 7 days, showed that DPSCs grew stably after CL treatment. The ICP-AES analysis determined the concentration of calcium in CL **(E)**. CAP: calcium phosphate lipid solution.

The spherical characteristic of CL could be observed under SEM (Figure 2), the particles dispersed better in PBS (Figure 2B) than DD water, and most of the particles attracted each other and formed larger granules in latter dilution (Figure 2C). The diameters ranged from 100 to 500 nm in DD water, average value was 204.3 nm. The concentration of calcium in the DD water was 2.06 ppm, rising to 2.19 ppm after 24 h. To simulate the body fluid condition, the CL was diluted with PBS, and calcium concentration was 3.02 ppm, but the difference was not significant (Figure 2E). The gradient solutions of CL were used to culture the DPSCs, to examine the biocompatibility. The results of CCK-8 showed that CL had no cellular toxicity (Figure 2D).

### Effects of CL on Regulating the Inflammatory Cytokines Expression of iDPSCs

**Figure 3:**
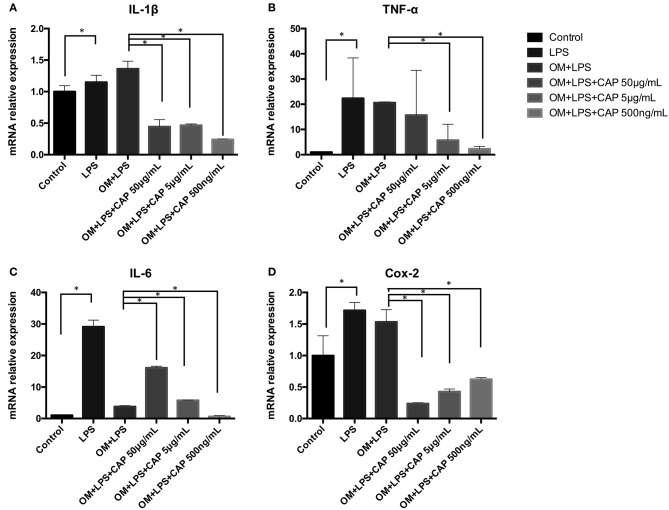
Quantitative PCR analysis results for the inflammatory cytokines *IL-1*β **(A)**, *TNF-*α **(B)**, *IL-6* **(C)**, and *COX-2* **(D)** upon treatment with CL. The asterisks indicate significant differences between groups (*p ≤ 0.05 one-way ANOVA and LSD comparison test) and the bars represent the standard deviation of three replicates.

Results of qPCR showed mRNA expression levels of all inflammatory cytokines were up-regulated in iDPSCs. CL had an obvious effect in down-regulating the expression of *IL-1*β*, TNF-*α, and *IL-6*, with negative correlation of the concentration, while suppressing the expression of *COX-2* dependent with the concentration (Figure 3).

### Effects of CL on the Osteo/Odontogenic Differentiation of iDPSCs

**Figure 4:**
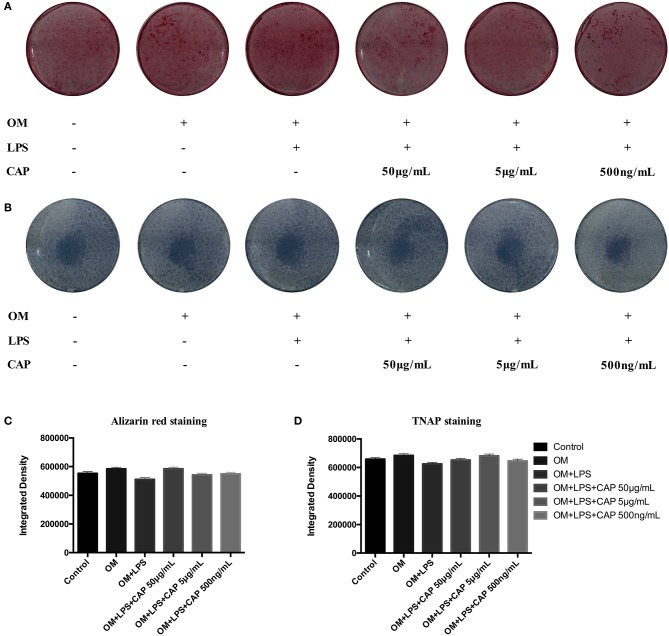
The results of Alizarin red staining **(A)** and TNAP staining assay **(B)** showed the odontogenic ability of CL, and the results of quantitative analysis **(C,D)** showed the differences were not significant (*p* > 0.05). OM represented osteogenic medium.

**Figure 5:**
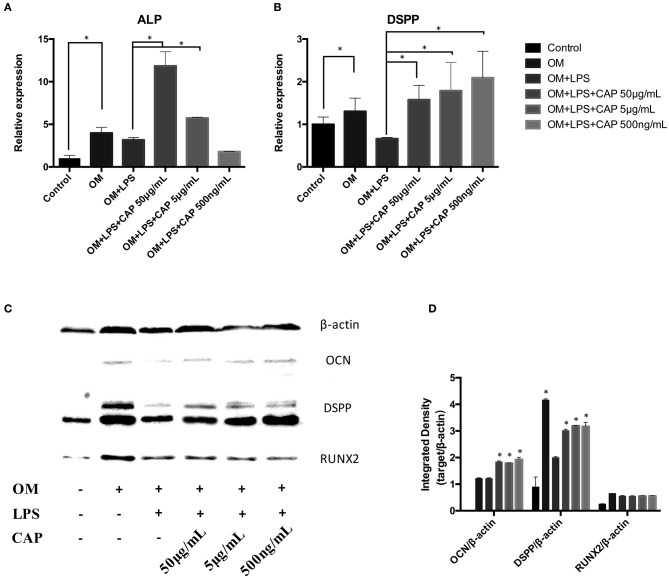
Quantitative PCR analysis of the odontoblast differentiation markers *ALP* **(A)** and *DSPP* **(B)**, and western blot analysis of osteo/odontogenic markers **(C)**. The integrated density of bands were analyzed by ImageJ **(D)**. The molecular weights of marked protein were listed followed: β-actin 45kD, OCN 50-55kD, DSPP 37-40kD, RUNX2 55-62kD. The asterisks indicate significant differences between groups (*p ≤ 0.05 one-way ANOVA and LSD comparison test) and the bars represent the standard deviation of three replicates.

The mineralization potential of iDPSCs under CL treatment was examined through Alizarin red staining (Figures 4A–C) and TNAP staining assay (Figures 4B–D). Compared to DPSCs cultured in the osteogenic medium (OM), less mineralized nodules formed in cultured iDPSCs, but CL could significantly induce the formation of calcium compounds in iDPSCs. The ALP activity varied in the same trend, but the differences were not significant (Figure 4D). The osteo/odontogenic differentiation ability was further investigated by examining related biomarkers and their downstream proteins. The qPCR analysis showed that CL significantly increased the expression of ALP and DSPP at mRNA level (*p* < 0.05) (Figures 5A,B), the expression of ALP was up-regulated most under 50 μg/mL (Figure 5A), while DSPP increased most under 500 ng/mL (Figure 5B). CL significantly increased the expression of DSPP and OCN at protein level, but the expression of RUNX2 showed no significance (Figures 5C,D, *p* > 0.05). This result was consistent with previous research, showing DSPP and RUNX2 expressed at the different stages of tooth development (Chen et al., 2009).

## Discussion

Calcium-phosphate lipid is formulated using a reverse micro-emulsion system. In separate round-bottom flasks, water droplets containing either calcium or phosphate are stirred in an oil phase containing cyclohexane and the surfactant Igepal. When the two emulsions are mixed and the separate droplets collide, the soluble calcium and phosphate react to form nanoprecipitates, still suspended in the water droplets. The final nanoparticle is generated by adding free lipids to the cores (Satterlee and Huang, 2016). The formation of asymmetric bilayer occurs when organic solvent is removed from the mixture and exposed to an aqueous solution. The inner leaflet is a cationic lipid to encapsulate negatively charged polynucleotides, and neutral lipid is placed on the outer leaflet to decrease non-specific cellular uptake/toxicity (Li et al., 2012). Till now, calcium-phosphate lipid has been widely considered as a carrier, however, its cellular compatibility has been barely studied. In this study, CL was demonstrated as anti-inflammatory and osteo/odontogenic to iDPSCs, which could be a potential material for vital pulp therapy (VPT).

Lipid mediators are widely appreciated for their important roles in initiating the leukocyte traffic required in host defense (Cotran et al., 1999). Novel families of lipid mediators could actively stimulate cessation of leukocytic infiltration, counterregulation of pro-inflammatory mediators, and the uptake of apoptotic neutrophils and cellular debris (Serhan et al., 2014), probably via cell-cell interactions within inflammatory exudates (i.e., pus), controlling the size, magnitude, and duration of the inflammatory event (Serhan, 2007). It had been demonstrated that specialized lipid mediators which were biosynthesized during self-limited acute inflammatory response (Serhan et al., 2014), called RvD1, RvD5, and PD1, could directly enhance phagocytosis of *E. coli*, counter-regulate a panel of pro-inflammatory genes, including NF-κB and TNF-α, and cooperate with antimicrobial process (Chiang et al., 2012; Jia et al., 2019). In the so-called lipid-mediator class switching process, specialized pro-resolving mediators (SPMs) were produced via a prostaglandins and leukotrienes (LTs) switch. SPMs triggered multiple reactions, such as limiting neutrophil tissue infiltration and influencing the translation of mRNAs encoding enzymes, and they were isolated in low-dose and locally active (Serhan et al., 2011), resulting in reducing exudate IL-6 and human neutrophil trans-endothelial migration in response to TNF-α (Dalli et al., 2013). Calcium-phosphate lipid might be able to trigger this reaction due to its similar lipid-bilayer structure, to rescue the inflammatory condition, which was consistent with the results that low-dose CL showed more efficient reduction in IL-1β, IL-6, and TNF-α (Figures 3A–C). Further mechanisms need to be studied in future research, including macrophage reactions and COX-2 related pathway.

Because calcium-phosphate is acid-sensitive (Ivanets et al., 2019), after the CL is delivered to the cellular endosome, the late endosome's acidic environment will dissolve the calcium-phosphate core (Li et al., 2010). It had been proved that calcium ions of calcium-phosphate lipid released more with a lower pH (Tang et al., 2015). In our study, the pH of DD water was about 7.7 and was freshly prepared (data not shown), with the pH a little bit higher than PBS (pH = 7.4). Calcium released more in PBS (Figure 2E), which was consistent with previous conclusions. Since Ca^2+^ is one of the critical factors in cell differentiation (Maeno et al., 2005; Valerio et al., 2009; Rahmanian et al., 2019), mineralization (Dvorak et al., 2014), and activation of multiple cellular pathways (Yang et al., 2017; Liu et al., 2019), CL might influence iDPSCs differentiation via free Ca^2+^ released. Also, it was reported that lipid membrane behavior upon local application of Ca^2+^ might contribute to several cellular processes, such as cell division and trafficking of proteins (Ali Doosti et al., 2017), and cationic liposomes were reported to be able to inhibit the activation of phosphatidylinositol-3 kinase-Akt (PI3K-Akt) pathway (Inoh et al., 2017). CL was supposed to enhance osteo/odontogenesis for the richness of Ca^2+^; upstream and downstream pathways still need to be further studied.

## Conclusions

In the present work, a widely studied vector, calcium-phosphate lipid (CL), was proved to suppress the expression of inflammatory cytokines and promote the osteo/odontogenic differentiation of iDPSCs, demonstrating itself as a biocompatible and anti-inflammatory material. CL has a broad application prospect for its bioactivity and flexible physical property, and thus represents a promising pulpal regeneration material to improve residual dental pulp precursor cells' response. Detailed research of the *in-vivo* biological properties of this material are needed to guide its future clinical applications.

## Data Availability Statement

All datasets generated for this study are included in the article/supplementary material.

## Ethics Statement

The studies involving human participants were reviewed and approved by The Ethics Committee of the Peking University Health Science Center. The patients/participants provided their written informed consent to participate in this study.

## Author Contributions

YW put the hypothesis forward and approved the outcomes. ZL provided the technical support of material fabrication. NZ carried out the experiments with the assistance of DW and FX, then wrote the manuscript draft. MQ revised the final manuscript.

### Conflict of Interest

The authors declare that the research was conducted in the absence of any commercial or financial relationships that could be construed as a potential conflict of interest.

## References

[B1] Alexis F. Pridgen E. Molnar L. K. Farokhzad O. C. (2008). Factors affecting the clearance and biodistribution of polymeric nanoparticles. Mol. Pharm. 5, 505–515. 10.1021/mp800051m 18672949 PMC2663893

[B2] Ali Doosti B. Pezeshkian W. Bruhn D. S. Ipsen J. H. Khandelia H. Jeffries G. D. M.. (2017). Membrane tubulation in lipid vesicles triggered by the local application of calcium ions. Langmuir 33, 11010–11017. 10.1021/acs.langmuir.7b01461 28910109

[B3] Chen S. Gluhak-Heinrich J. Wang Y. H. Wu Y. M. Chuang H. H. Chen L.. (2009). Runx2, osx, and dspp in tooth development. J. Dent. Res. 88, 904–909. 10.1177/0022034509342873 19783797 PMC3045537

[B4] Chiang N. Fredman G. Bäckhed F. Oh S. F. Vickery T. Schmidt B. A.. (2012). Infection regulates pro-resolving mediators that lower antibiotic requirements. Nature 484, 524–528. 10.1038/nature11042 22538616 PMC3340015

[B5] Cotran R. S. Kumar V. Collins T. Robbins S. L. (1999). Robbins Pathologic Basis of Disease. Philadelphia: Saunders.

[B6] Dalli J. Zhu M. Vlasenko N. A. Deng B. Haeggstrom J. Z. Petasis N. A.. (2013). The novel 13S,14S-epoxy-maresin is converted by human macrophages to maresin 1 (MaR1), inhibits leukotriene A4 hydrolase (LTA4H), and shifts macrophage phenotype. FASEB J. 27, 2573–2583. 10.1096/fj.13-227728 23504711 PMC3688739

[B7] Dvorak M. Siddiqua A. Ward D. Carter D. Dallas S. (2014). Physiological changes in extracellular calcium concentration directly control osteoblast function in the absence of calciotropic hormones. Proc. Natl. Acad. Sci. U.S.A. 101, 5140–5145. 10.1073/pnas.0306141101 15051872 PMC387387

[B8] Fukushima T. Hayakawa T. Inoue Y. Miyazaki K. Okahata Y. (2004). Intercalation behavior and tensile strength of DNA–lipid films for the dental application. Biomaterials 25, 5491–5497. 10.1016/j.biomaterials.2004.01.006 15142730

[B9] Graham F. L. van der Eb A. J. (1973). A new technique for the assay of infectivity of human adenovirus 5 DNA. Virology 52, 456–467. 10.1016/0042-6822(73)90341-3 4705382

[B10] Inoh Y. Haneda A. Tadokoro S. Yokawa S. Furuno T. (2017). Cationic liposomes suppress intracellular calcium ion concentration increase via inhibition of PI3 kinase pathway in mast cells. Biochim Biophy Acta Biomembr. 1859, 2461–2466. 10.1016/j.bbamem.2017.09.025 28966111

[B11] Ivanets A. I. Shashkova I. L. Kitikova N. V. Maslova M. V. Mudruk N. V. (2019). New heterogeneous synthesis of mixed Ti-Ca-Mg phosphates as efficient sorbents of 137Cs, 90Sr and 60Co radionuclides. J Taiwan Inst Chem Engin. 104, 151–159. 10.1016/j.jtice.2019.09.001

[B12] Jia B. Ma Y. M. Liu B. Chen P. Hu Y. Zhang R. (2019). Synthesis, antimicrobial activity, structure-activity relationship, and molecular docking studies of indole diketopiperazine alkaloids. Front. Chem. 7:837. 10.3389/fchem.2019.00837 31850323 PMC6897290

[B13] Kord Forooshani P. Polega E. Thomson K. Bhuiyan M. S. A. Pinnaratip R. Trought M.. (2019). Antibacterial properties of mussel-inspired polydopamine coatings prepared by a simple two-step shaking-assisted method. Front. Chem. 7:631. 10.3389/fchem.2019.00631 31608272 PMC6773806

[B14] Li J. Chen Y.-C. Tseng Y.-C. Mozumdar S. Huang L. (2010). Biodegradable calcium phosphate nanoparticle with lipid coating for systemic siRNA delivery. J. Control. Release 142, 416–421. 10.1016/j.jconrel.2009.11.008 19919845 PMC2833237

[B15] Li J. Yang Y. Huang L. (2012). Calcium phosphate nanoparticles with an asymmetric lipid bilayer coating for siRNA delivery to the tumor. J. Control. Release 158, 108–114. 10.1016/j.jconrel.2011.10.020 22056915 PMC3288896

[B16] Li L. Tian F. Chang H. Zhang J. Wang C. Rao W.. (2019). Interactions of bacteria with monolithic lateral silicon nanospikes inside a microfluidic channel. Front. Chem. 7:483. 10.3389/fchem.2019.00483 31355180 PMC6640657

[B17] Liu Q. Li N. Qiao Z. Li W. Wang L. Zhu S.. (2019). The multiple promotion effects of ammonium phosphate-modified Ag3PO4 on photocatalytic performance. Front. Chem 7:866. 10.3389/fchem.2019.00866 31921784 PMC6937216

[B18] Maeno S. Niki Y. Matsumoto H. Morioka H. Yatabe T. Funayama A.. (2005). The effect of calcium ion concentration on osteoblast viability, proliferation and differentiation in monolayer and 3D culture. Biomaterials 26, 4847–4855. 10.1016/j.biomaterials.2005.01.006 15763264

[B19] Maitra A. (2005). Calcium phosphate nanoparticles: second-generation nonviral vectors in gene therapy. Expert Rev. Mol. Diagn. 5, 893–905. 10.1586/14737159.5.6.893 16255631

[B20] Nuti S. Fernández-Lodeiro J. Del Secco B. Rampazzo E. Rodríguez-González B. Capelo J. L.. (2018). Engineered nanostructured materials for ofloxacin delivery. Front. Chem. 6:554. 10.3389/fchem.2018.00554 30538980 PMC6277636

[B21] Rahmanian M. seyfoori A. Dehghan M. M. Eini L. Naghib S. M. Gholami H. (2019). Multifunctional gelatin–tricalcium phosphate porous nanocomposite scaffolds for tissue engineering and local drug delivery: *in vitro* and *in vivo* studies. J. Taiwan Inst. Chem. Engin. 101, 214–220. 10.1016/j.jtice.2019.04.028

[B22] Satterlee A. B. Huang L. (2016). Current and future theranostic applications of the lipid-calcium-phosphate nanoparticle platform. Theranostics 6, 918–929. 10.7150/thno.14689 27217828 PMC4876619

[B23] Serhan C. N. (2007). Resolution phase of inflammation: novel endogenous anti-inflammatory and proresolving lipid mediators and pathways. Annu. Rev. Immunol. 25, 101–137. 10.1146/annurev.immunol.25.022106.141647 17090225

[B24] Serhan C. N. Chiang N. Dalli J. Levy B. D. (2014). Lipid mediators in the resolution of inflammation. Cold Spring Harb. Perspect. Biol. 7, a016311–a016311. 10.1101/cshperspect.a016311 25359497 PMC4315926

[B25] Serhan C. N. Fredman G. Yang R. Karamnov S. Belayev L. S. Bazan N. G.. (2011). Novel proresolving aspirin-triggered DHA pathway. Chem. Biol. 18, 976–987. 10.1016/j.chembiol.2011.06.008 21867913 PMC3164791

[B26] Sokolova V. V. Radtke I. Heumann R. Epple M. (2006). Effective transfection of cells with multi-shell calcium phosphate-DNA nanoparticles. Biomaterials 27, 3147–3153. 10.1016/j.biomaterials.2005.12.030 16469375

[B27] Tang J. Li L. Howard C. B. Mahler S. M. Huang L. Xu Z. P. (2015). Preparation of optimized lipid-coated calcium phosphate nanoparticles for enhanced *in vitro* gene delivery to breast cancer cells. J. Mater. Chem. B 3, 6805–6812. 10.1039/C5TB00912J 27213045 PMC4869335

[B28] Tyo A. Welch S. Hennenfent M. Kord Fooroshani P. Lee B. P. Rajachar R. (2019). Development and characterization of an antimicrobial polydopamine coating for conservation of humpback whales. Front. Chem. 7:618. 10.3389/fchem.2019.00618 31620421 PMC6759777

[B29] Valerio P. Pereira M. M. Goes A. M. Leite M. F. (2009). Effects of extracellular calcium concentration on the glutamate release by bioactive glass (BG60S) preincubated osteoblasts. Biomed. Mater. 4:045011. 10.1088/1748-6041/4/4/045011 19636109

[B30] Verderosa A. D. Totsika M. Fairfull-Smith K. E. (2019). Bacterial biofilm eradication agents: a current review. Front. Chem. 7:824. 10.3389/fchem.2019.00824 31850313 PMC6893625

[B31] Wang Y. Zhao Y. Jia W. Yang J. Ge L. (2013). Preliminary study on dental pulp stem cell-mediated pulp regeneration in canine immature permanent teeth. J. Endod. 39, 195–201. 10.1016/j.joen.2012.10.002 23321230

[B32] Williams D. E. Grant K. B. (2019). Metal-assisted hydrolysis reactions involving lipids: a review. Front. Chem. 7:14. 10.3389/fchem.2019.00014 30838196 PMC6390409

[B33] Xu H. Yang D. Jiang D. Chen H.-Y. (2019). Phosphate assay kit in one cell for electrochemical detection of intracellular phosphate ions at single cells. Front. Chem. 7:360. 10.3389/fchem.2019.00360 31179270 PMC6542946

[B34] Xu Z. Ramishetti S. Tseng Y.-C. Guo S. Wang Y. Huang L. (2013). Multifunctional nanoparticles co-delivering Trp2 peptide and CpG adjuvant induce potent cytotoxic T-lymphocyte response against melanoma and its lung metastasis. J. Control. Release 172, 259–265. 10.1016/j.jconrel.2013.08.021 24004885

[B35] Yang S. Sun H. Yan J. Guo X. Li D. Zhou D. (2017). Persistent mechanical stretch-induced calcium overload and MAPK signal activation contributed to SCF reduction in colonic smooth muscle *in vivo* and *in vitro* AU - Dong, Fang. J. Recept. Signal Transduct. 37, 141–148. 10.1080/10799893.2016.1203939 27400729

[B36] Yang Y. Hu Y. Wang Y. Li J. Liu F. Huang L. (2012). Nanoparticle delivery of pooled siRNA for effective treatment of non-small cell lung cancer. Mol. Pharm. 9, 2280–2289. 10.1021/mp300152v 22686936 PMC3484223

[B37] Yao J. Zhang Y. Ramishetti S. Wang Y. Huang L. (2013). Turning an antiviral into an anticancer drug: nanoparticle delivery of acyclovir monophosphate. J. Control. Release 170, 414–420. 10.1016/j.jconrel.2013.06.009 23791977 PMC3742590

[B38] Zhai Y. Wang Y. Rao N. Li J. Li X. Fang T.. (2019). Activation and biological properties of human beta defensin 4 in stem cells derived from human exfoliated deciduous teeth. Front. Physiol. 10:1304. 10.3389/fphys.2019.01304 31695620 PMC6817489

[B39] Zhang J. Miao L. Guo S. Zhang Y. Zhang L. Satterlee A.. (2014). Synergistic anti-tumor effects of combined gemcitabine and cisplatin nanoparticles in a stroma-rich bladder carcinoma model. J. Control. Release 182, 90–96. 10.1016/j.jconrel.2014.03.016 24637468 PMC4009696

[B40] Zhi D. Bai Y. Yang J. Cui S. Zhao Y. Chen H.. (2018). A review on cationic lipids with different linkers for gene delivery. Adv. Colloid Interface Sci. 253, 117–140. 10.1016/j.cis.2017.12.006 29454463

[B41] Zhu N. Chatzistavrou X. Ge L. Qin M. Papagerakis P. Wang Y. (2019). Biological properties of modified bioactive glass on dental pulp cells. J. Dent. 83, 18–26. 10.1016/j.jdent.2019.01.017 30776406

